# Controlled Release of Lysozyme from Double-Walled Poly(Lactide-*Co*-Glycolide) (PLGA) Microspheres

**DOI:** 10.3390/polym9100485

**Published:** 2017-10-03

**Authors:** Rezaul H. Ansary, Mokhlesur M. Rahman, Nasir Mohamad, Tengku M. Arrif, Ahmad Zubaidi A. Latif, Haliza Katas, Wan Sani B. Wan Nik, Mohamed B. Awang

**Affiliations:** 1 Faculty of Pharmacy, International Islamic University Malaysia, Kuantan 25200, Malaysia; ansarychem@gmail.com; 2 Department of Chemistry, University of Rajshahi, Rajshahi 6205, Bangladesh; 3 Institute for Community Development & Quality of Life (i-CODE), Universiti Sultan Zainal Abidin, Kuala Nerus 21300, Terengganu, Malaysia; nasirmohamad@unisza.edu.my (N.M.); tg_mariff@unisza.edu.my (T.M.A.); 4 Faculty of Medicine, Universiti Sultan Zainal Abidin, Kuala Nerus 21300, Terengganu, Malaysia; azubaidi@unisza.edu.my; 5 Faculty of Pharmacy, Universiti Kebangsaan Malaysia, Kuala Lumpur 50300, Malaysia; hliza@pharmacy.ukm.my; 6 School of Ocean Engineering, Universiti Malaysia Terengganu, 21300 Kuala Nerus, Terengganu, Malaysia; niksani@umt.edu.my; 7 Faculty of Pharmacy, Cyberjaya University College of Medical Sciences, Cyberjaya 63000, Malaysia; mohamedawang@cybermed.edu.my

**Keywords:** drug delivery, controlled release, double-walled microspheres, poly(lactide-*co*-glycolide), therapeutic proteins

## Abstract

Double-walled microspheres based on poly(lactide-*co*-glycolide) (PLGA) are potential delivery systems for reducing a very high initial burst release of encapsulated protein and peptide drugs. In this study, double-walled microspheres made of glucose core, hydroxyl-terminated poly(lactide-*co*-glycolide) (Glu-PLGA), and carboxyl-terminated PLGA were fabricated using a modified water-in-oil-in-oil-in-water (w_1_/o/o/w_2_) emulsion solvent evaporation technique for the controlled release of a model protein, lysozyme. Microspheres size, morphology, encapsulation efficiency, lysozyme in vitro release profiles, bioactivity, and structural integrity, were evaluated. Scanning electron microscopy (SEM) images revealed that double-walled microspheres comprising of Glu-PLGA and PLGA with a mass ratio of 1:1 have a spherical shape and smooth surfaces. A statistically significant increase in the encapsulation efficiency (82.52 ± 3.28%) was achieved when 1% (*w*/*v*) polyvinyl alcohol (PVA) and 2.5% (*w*/*v*) trehalose were incorporated in the internal and external aqueous phase, respectively, during emulsification. Double-walled microspheres prepared together with excipients (PVA and trehalose) showed a better control release of lysozyme. The released lysozyme was fully bioactive, and its structural integrity was slightly affected during microspheres fabrication and in vitro release studies. Therefore, double-walled microspheres made of Glu-PLGA and PLGA together with excipients (PVA and trehalose) provide a controlled and sustained release for lysozyme.

## 1. Introduction

In recent years, controlled-release formulations of the therapeutic protein and peptide drugs using biodegradable polymeric devices have attracted much attention in research community around the world [1,2,3,4]. Microspheres and nanoparticles based biodegradable polymeric drug delivery systems offer an easy administration of therapeutic protein and peptide drugs, including oral, pulmonary, and parenteral injection [5,6,7]. The polymeric devices guard the encapsulated drug against degradation and control its site-specific release. Among the different classes of biodegradable polymers, poly(d,l-lactide-*co*-glycolide) (PLGA) and poly(d,l-lactide) (PLA) are the first synthetic polymers that have been approved by the Food and Drug Administration (FDA) for drug delivery [8]. PLGA and PLA undergo hydrolysis in the body to produce biodegradable metabolite monomers, lactic acid, and glycolic acid, which are readily eliminated from the body through the Krebs cycle [9]. Due to their biodegradable characteristics, these polymers have the advantage of not requiring surgery for removal from the body upon the completion of drug release. However, monolithic or single-walled PLGA microspheres and nanoparticles have several intrinsic limitations in a controlled and sustained release of high water-soluble protein and peptide drugs along with their inherent stability problem. They include the high initial burst followed by the prolonged and incomplete release that results in difficulty in achieving zero-order release and a low encapsulation efficiency [10,11,12]. In double-walled microspheres, drug encapsulated in the interior cover is surrounded by a drug-free outer layer that overcomes most of the problems stated above [13,14,15]. In this case, the drug-free outer layer acts as an additional diffusion barrier inhibiting the drug from leaking out during the microspheres fabrication process. Moreover, an appropriate selection of the inner and outer polymer materials, and the layer thickness control the drug release rates [16,17]. Many researchers have developed methods to fabricate microspheres with a double or multi-layered structure from polymer blends, including dip- or pan-coating process [18], fluidized bed technology [19], spray drying [20], a precision particle fabrication technology [21,22], and emulsion solvent evaporation [23,24,25]. Based on the core/shell phenomenon, a one-step water-in-oil-in-oil-in-water (w_1_/o/o/w_2_) technique has been currently developed for the fabrication of double-walled microspheres loaded with hydrophilic protein and peptide drugs [23,24]. In this study, a modified water-in-oil-in-oil-in-water (w_1_/o/o/w_2_) solvent evaporation technique was used to produce a model protein-lysozyme encapsulated double-walled microspheres from a polymer blend of fast degrading glucose core, hydroxyl-terminated PLGA (Glu-PLGA), and other commonly used moderate degrading carboxyl-terminated PLGA polymers. Single-walled microspheres comprising of Glu-PLGA and PLGA were also prepared to show the effectiveness of blending of two different PLGAs in reducing the high initial burst. Moreover, double-walled microspheres were prepared together with excipients (PVA and trehalose) to investigate the phenomena of lysozyme denaturation during microspheres fabrication.

## 2. Materials and Methods

### 2.1. Materials

Poly(lactide-*co*-glycolide) (PLGA star, glucose core, hydroxyl terminated, with a ratio LA:GA as 1:1) (Glu-PLGA) average *M*_n_ 15,000 and 50,000, polyvinyl alcohol (PVA; *M*_w_ 30,000–70,000; 87–90% hydrolyzed), chicken egg-white lysozyme, bicinchoninic acid kit (BCA1-1KT), micrococcus lysodeikticus (ATCC 4698), and dimethyl sulfoxide (DMSO) were purchased from Sigma-Aldrich (St. Louis, MO, USA). Ethyl acetate (EA) and dichloromethane (DCM) were purchased from Fisher Scientific (Leicestershire, UK). PLGA (50:50 with an intrinsic viscosity 0.4 dL/g) were obtained from Purac (Gorinchem, The Netherlands). All of the other chemicals and solvents used in this study were of analytical grade.

### 2.2. Methods

#### 2.2.1. Preparation of Microspheres

A previously developed water-in-oil-in-oil-in-water (w_1_/o/o/w_2_) emulsion solvent evaporation method was modified and employed to fabricate lysozyme encapsulated in double-walled microspheres [23]. A detailed method of preparation can be found in our previous publications [26,27]. Briefly, PLGA was dissolved in ethyl acetate (EA) and emulsified with an aqueous solution of lysozyme using a basic homogenizer of IKA^®^ T 10 (Werke GmbH and Co., Staufen, Germany). The obtained water-in-oil (w_1_/o) emulsion was then poured into dichloromethane (DCM) solution of Glu-PLGA. The resulting two phases were homogenized using the same homogenizer. The water-in-oil-in-oil (w_1_/o/o) emulsion was formed which was subsequently transferred to an external aqueous phase containing 1% (*w*/*v*) PVA solution. In this step, water-in-oil-in-oil-in-water (w_1_/o/o/w_2_) emulsion was formed after homogenization using the same homogenizer. The resultant w_1_/o/o/w_2_ emulsion was then added to an aqueous 0.5% (*w*/*v*) PVA solution and stirred magnetically at room temperature and pressure was reduced to evaporate dichloromethane and ethyl acetate. The hardened microspheres in suspension were then centrifuged and washed with double distilled water to remove the excess PVA. Finally, the microspheres were freeze-dried overnight using a freeze-dryer (Alpha 1-4 LD model, Christ Co., Berlin, Germany) and stored at −20 °C until further characterization. In addition, conventional water-in-oil-in-water (w_1_/o/w_2_) emulsion solvent evaporation technique was used to fabricate single-walled microspheres of Glu-PLGA and PLGA for comparison. Table 1 shows the details of polymers and solvents that were used for microspheres fabricated in different formulations.

#### 2.2.2. Characterization of Lysozyme Loaded Microspheres

##### Particle Size Analysis

The particle size of the hardened microspheres was determined by laser diffraction using a Laser Particle Size Analyzer BT-9300H (Dandong Bettersize Instruments, Dandong, China). Prior to freeze-drying, an aliquot of the wet microspheres was employed to measure the particle size, which is used to estimatethe volume median diameter (*D* 50%). Particle size distribution (polydispersity) was expressed by the span value, which is calculated as follows:

(1) $$\mathrm{Span}=\frac{D_{90\%}{-D}_{10\%}}{D_{50\%}},$$

where, *D*_10%_, *D*_50%_, and *D*_90%_ are the particle diameters at 10%, 50%, and 90% cumulative volume, respectively, and the cumulative percentage (%) is computed by summing the percentages of particles smaller than their equivalent diameter.

##### Microspheres Surface Morphology

A Scanning Electron Microscope (SEM) (Carl Zeiss Evo^®^ 50, Oberkochen, Germany) was used to observe the shape and surface morphologies of the microspheres. Freeze-dried microspheres were first mounted onto metal stubs using double-sided adhesive tape. The stubs were then coated with a thin layer of gold under vacuum using a sputter coater. The microspheres were imaged using SEM with an accelerating voltage set to 5–20 KV.

##### Determining Microspheres Polymer Orientation

The selective dissolution technique developed by Lee et al. (2002) was employed to explore the core and shell polymer layer of double-walled microspheres comprising Glu-PLGA and PLGA [13]. In this study, different solubilities of Glu-PLGA and PLGA in ethyl acetate were used to recognize the core and shell polymer layer. A detailed method can be found in the previous articles [26,27].

##### Lysozyme Encapsulation Efficiency

In this study, micro-BCA protein assay method developed by Sah (1997) was used to quantify lysozyme in the microspheres [28]. Freeze-dried microspheres of approximately 10 mg were dissolved in 1 mL DMSO and incubated for 1 h at room temperature. After complete digestion, the solution was mixed with 5 mL of 0.05 M NaOH solution containing 0.5% (*w*/*v*) SDS (sodium dodecyl sulfate). A micro-BCA protein assay kit was used to quantify lysozyme in the clear solution. In this method, empty microspheres (lysozyme free) were also used as a control. The following equations were used to estimate lysozyme loading and encapsulation efficiency.

(2) $$\mathrm{Actual}\;\mathrm{drug}\;\mathrm{loading}\;\left(\%\right)=\frac{\mathrm{Encapsulated}\;\mathrm{amount}\;\mathrm{of}\;\mathrm{lysozyme}}{\mathrm{Total}\;\mathrm{dry}\;\mathrm{weight}\;\mathrm{of}\;\mathrm{microspheres}}\times 100\%$$

(3) $$\mathrm{Encapsulation}\;\mathrm{efficiency}\;\left(\%\right)=\frac{\mathrm{Encapsulated}\;\mathrm{amount}\;\mathrm{of}\;\mathrm{lysozyme}}{\mathrm{Total}\;\mathrm{amount}\;\mathrm{of}\;\mathrm{lysozyme}\;\mathrm{used}\;\mathrm{for}\;\mathrm{encapsulation}}\times 100\%$$

##### Microspheres Yield

At the end of microspheres fabrication, lyophilized microspheres were weighed and the yield was measured by:

(4) $$\mathrm{Microspheres}\;\mathrm{yield}\;\left(\%\right)=\frac{\mathrm{Amount}\;\mathrm{of}\;\mathrm{recovered}\;\mathrm{microspheres}}{\mathrm{Total}\;\mathrm{amount}\;\mathrm{of}\;\mathrm{polymer}\;\mathrm{and}\;\mathrm{drug}\;\mathrm{used}\;\mathrm{initially}\;}\times 100\%$$

##### In Vitro Release Studies

To study the in vitro release profiles, approximately 10 mg lyophilized microspheres were suspended in 1.5 mL phosphate buffer saline (pH 7.4) containing Eppendorf tubes. Sodium azide (NaN_3_) aqueous solution (0.01%, *w*/*v*) was added in the tubes as a bacteriostatic agent. The tubes were placed in an incubator shaker and agitated horizontally at 100 rpm with 37 °C. At specific time intervals, the tubes were centrifuged and the supernatant was withdrawn completely. A constant pH sink condition was maintained throughout the release studies as an equal volume of fresh buffer was poured after each sampling. A micro-BCA protein assay kit was used to quantify the lysozyme level in the supernatant. Each of the samples was analyzed in triplicate.

##### Lysozyme Bioactivity Measurements

Since lysozyme hydrolyses the bacterial cell walls, *Micrococcus lysodeikticus* was used as the substrate to determine the enzymatic activity of lysozyme released from the microspheres. The bioactivity of lysozyme was calculated by determining the rate of the decrease in turbidity of the cell suspension. To accomplish this, a 0.2 mg/mL suspension of *Micrococcus lysodeikticus*was prepared in 66 mM phosphate buffer with a pH 6.2 (7.59 g NaH_2_PO_4_·H_2_O, 1.96 g Na_2_HPO_4_·2H_2_O). For measuring the turbimetric rate, 10 µL aliquot of the day 1 released lysozyme sample (concentration adjusted to 50 µg/mL) was taken in a cuvette and mixed with 1.3 mL substrate suspension. The cuvette was immediately placed in a spectrophotometer (Shimadzu UV-1800, Shimadzu Corporation, Kyoto, Japan). The decrease in the turbidity of the suspension was measured for 3 min at 450 nm. The following equation was used to calculate the bioactivity of released lysozyme [29,30].

(5) $$\mathrm{Bioactivity}\left(\%\right)=\frac{∆Abs_{3\min}\left(\mathrm{Actual}\right)}{∆Abs_{3\min}\left(\mathrm{Theoretical}\right)}\times 100\%$$

#### 2.2.3. Polyacrylamide Gel Electrophoresis (SDS-PAGE)

A non-reducing SDS-PAGE was performed to determine the possible degradation or aggregation of lysozyme during microspheres fabrication and in vitro release studies. In this case, lysozyme encapsulated microspheres as prepared in presence of 1% PVA (*w*/*v*) in internal aqueous phase and 2.5% trehalose (*w*/*v*) in the external aqueous phase was examined. The extracted and released lysozyme was mixed with a sample buffer (0.5 M Tris-HCl, pH 6.8, 25% glycerol, 2% SDS, 0.01% Bromophenol blue). The buffer to sample ratio of mixing was maintained as 1:4 (*v*/*v*). The samples were warmed for 5 min at 95 °C prior to loading. The process of electrophoresis was performed at a constant voltage of 200 V in a running buffer (0.3% (*w*/*v*) Tris base, 1.4% (*w*/*v*) glycine, 0.1% (*w*/*v*) SDS, pH 8.3) using a Bio-Red Mini-Protean electrophoresis system. The gels were stained with Coomassie blue to visualize the protein bands. The molecular weights of the visualized bands were compared to a protein molecular weight marker (3.5–260 kDa, Novex^®^ Sharp Pre-Stained). Native lysozyme treated under the same condition was used as a control.

### 2.3. Statistical Analysis

The results were expressed as the mean value ± standard deviation for three separate measurements. One-way analysis of variance (ANOVA), followed by *post hoc* Tukey multiple comparison tests (SPSS Inc., Chicago, IL, USA) were used to assess the statistical difference. A statistically significant difference was considered when the *p* value was less than 0.05.

## 3. Results and Discussion

### 3.1. Particle Size and Size Distribution of Microspheres

Microspheres mean particle size and size distributions (span value) are shown in Table 2. Double-walled microspheres made of Glu-PLGA and PLGA (1:1) in formulations LF1 and LF3 exhibited the mean particle size of 1.64 ± 0.58 µm and 2.65 ± 0.45 µm, respectively. In contrast, a larger mean particle size of 7.79 ± 1.03 µm and 8.04 ± 1.21 µm was observed in the formulation of LF2 and LF4 when the mass ratio of Glu-PLGA to PLGA was increased to 2:1. This statistically significant increase in mean particle size (*p* < 0.05) is consistent with the previous results [26,27]. Moreover, a statistically significant decrease in span value (*p* < 0.05) was observed in formulations LF1 and LF3 as compared to other double-walled and single-walled formulations. Polymer viscosity has a great influence on the mean size and span value of microspheres. The viscosity of polymer solution increases with increasing mass ratio of a polymer mixture, which resists emulsion droplets to be broken down into smaller droplets using the same shear stress [26,27]. Therefore, the larger emulsion droplets produce microspheres with slightly larger size. In addition, with an increasing polymer concentration, microspheres are produced at a faster rate with slightly larger size and size distribution.

### 3.2. Surface Morphology of Lysozyme Loaded Microspheres

**Figure 1:**
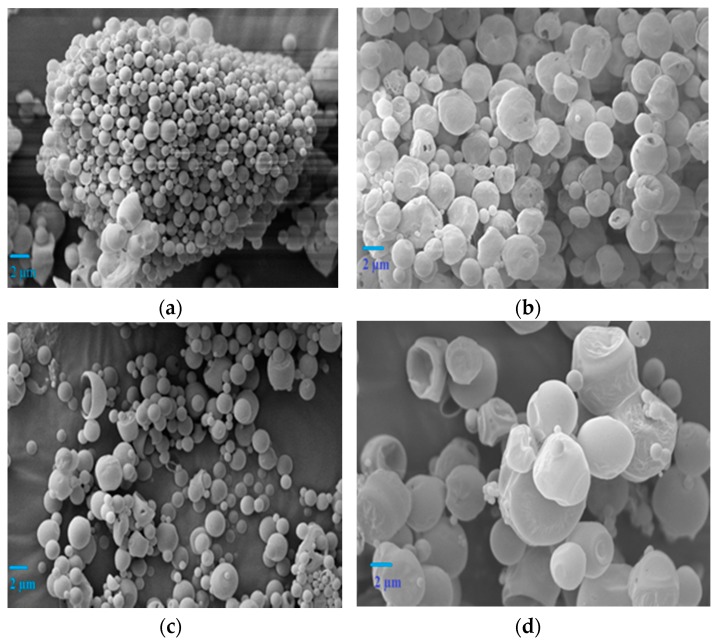
Scanning electron microscope (SEM) images of lysozyme loaded double-walled microspheres prepared in different formulations. (**a**) LF1; (**b**) LF2; (**c**) LF3; and, (**d**) LF4.

**Figure 2:**
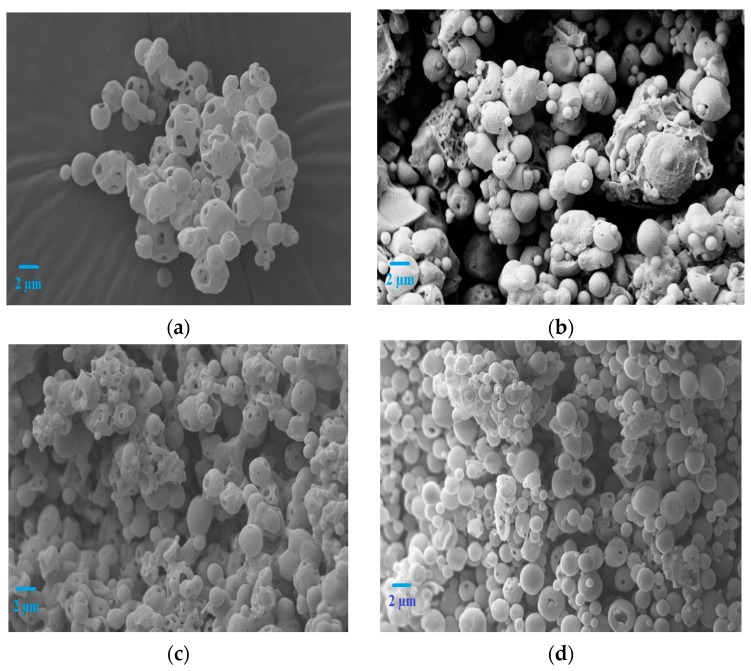
Scanning electron microscope (SEM) images of lysozyme loaded single-walled microspheres prepared in different formulations. (**a**) LF5; (**b**) LF6; (**c**) LF7; (**d**) LF8.

The surface morphologies (shape and size) of lysozyme loaded double-walled and single-walled microspheres were studied by SEM. The SEM analysis demonstrated that the double-walled microspheres made of Glu-PLGA and PLGA in a mass ratio of 1:1 were spherical in shape, smooth, and non-porous surfaces (Figure 1a,c). In contrast, increasing the mass ratio of Glu-PLGA to PLGA from 1:1 to 2:1 in formulations LF2 and LF4 resulted in microspheres with a deformed shape, non-porous surfaces. Moreover, some extent of roughness was observed on the surfaces of the microspheres (Figure 1b,d). The increased viscosity of the disperse phase with an increasing polymer concentration leads to fast polymer precipitation that results in microspheres with a deformed shape and uneven surfaces. On the other hand, single-walled microspheres of formulations LF5, LF7, and LF8 were highly porous, as illustrated in Figure 2a,c,d. The surface porosity of single-walled microspheres prepared in formulation LF6 was reduced (Figure 2b), as the polymer concentration of this formulation was doubled when compared to other single-walled formulations. Although the same amount of polymer (100 mg) corresponding to LF1 and LF3 was used in single-walled formulation LF6, the SEM analysis demonstrated that the surface morphologies of the microspheres prepared in double-walled formulations (LF1 and LF3) were significantly different from single-walled formulation LF6. The increased viscosity of the polymer solution might contribute to form coarse emulsion droplets that could be destabilized due to coalescence of the droplets resulting in the formation of larger size emulsion droplets. Therefore, the size and shape of the microspheres were beyond control due to the faster precipitation of the embryonic microspheres.

### 3.3. Microspheres Polymer Orientation Studies

The polymer (Glu-PLGA and PLGA) orientation in prepared microspheres was evaluated using a selective dissolution technique, as described in the previous publications, and found Glu-PLGA and PLGA as the core and shell layer, respectively [26,27].

### 3.4. Lysozyme Encapsulation Efficiency

The encapsulation efficiency and actual loading of lysozyme in double-walled and single-walled microspheres are shown in Table 2. The encapsulation efficiencies of double-walled microspheres prepared from Glu-PLGA and PLGA polymer in a mass ratio of 1:1 in formulations LF1 and LF3 were 71.04 ± 3.08% and 72.15 ± 3.54%, respectively. Increasing the mass ratio of Glu-PLGA to PLGA from 1:1 to 2:1 in formulations LF2 and LF4 enhanced the encapsulation efficiency to 82.84 ± 3.89% and 83.92 ± 4.66%, respectively. This statistically significant increase (*p* < 0.05) in the encapsulation efficiency of formulations LF2 and LF4 is assumed to be due to the increase in polymer viscosity with an increasing polymer mass. High viscous polymer-solution reduces the mobility of proteins in the emulsion droplets. Thus, the diffusion of hydrophilic lysozyme into the continuous aqueous phase might be reduced, which results in a higher encapsulation efficiency. Moreover, the polymer precipitation rate is faster at a higher polymer concentration, which in turn could slow down the diffusion rate of lysozyme from the emulsion droplets to the continuous phase, as the diffusion time is very short. Therefore, the increasing of polymer mass is considered to be the reason of slightly higher encapsulation efficiencies of formulations LF2 and LF4.

The encapsulation efficiencies of single-walled microspheres prepared in formulations LF5, LF6, LF7, and LF8 were calculated as 51.67 ± 3.56%, 62.03 ± 4.15%, 53.88 ± 2.97%, and 52.23 ± 3.90%, respectively. These single-walled formulations showed a statistically significant decrease (*p* < 0.05) in encapsulation efficiency when compared to double-walled formulations. SEM images of single-walled and double-walled formulations revealed that single-walled formulations were highly porous, whereas double-walled formulations were non-porous. The rapid diffusion of hydrophilic lysozyme from the disperse phase to the continuous phase is accounted for the formation of these pores. Therefore, single-walled formulations could not retard the rapid diffusion of lysozyme into the continuous phase, which resulted in low encapsulation efficiency. Although the same amount of polymer corresponding to formulations LF1 and LF3 was used in formulation LF6 (single-walled), the encapsulation efficiency of LF6 was not as high as LF1 and LF3. Lysozyme molecules encapsulated in the core of double-walled microspheres might be shielded from the rapid diffusion to the surrounding continuous phase due to the presence of another outer polymer layer. Single-walled microspheres cannot suppress the rapid diffusion of hydrophilic lysozyme to the external aqueous phase during emulsification and hardening, as they do not have any core/shell layer. Hence, lysozyme loaded double-walled microspheres showed a higher encapsulation efficiency as compared to single-walled microspheres.

### 3.5. In Vitro Lysozyme Release

**Figure 3:**
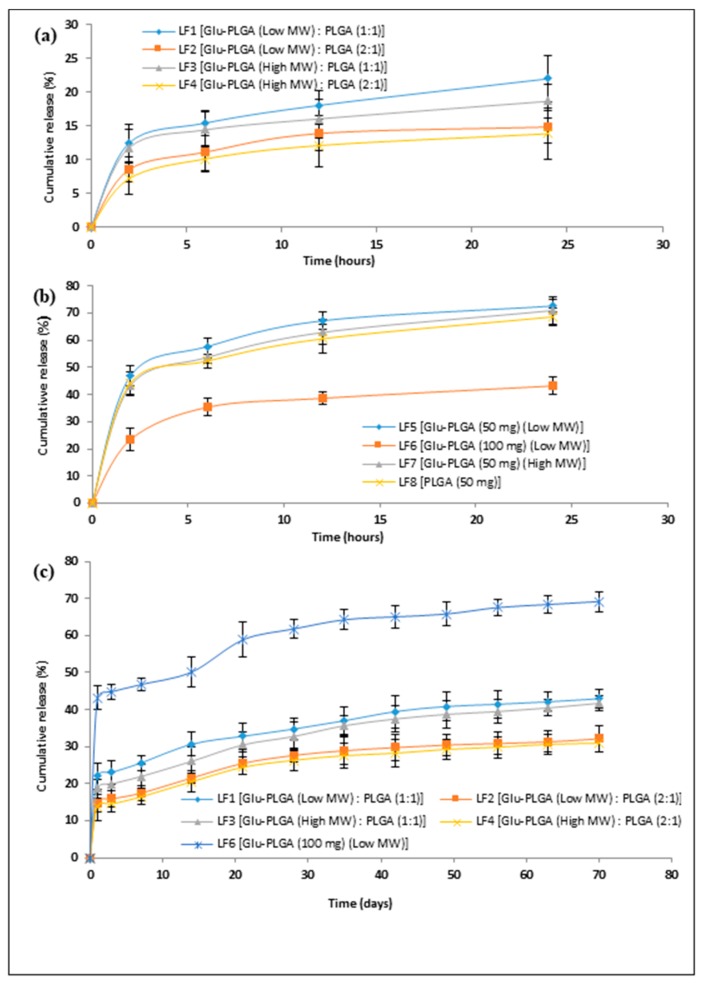
In vitro release profiles of lysozyme for (**a**) 24 h double-walled; (**b**) 24 h single-walled; and (**c**) 70 days double-walled and single-walled microspheres prepared in different formulations. The data are presented as mean ± SD (*n* = 3).

The in vitro release profiles of lysozyme from double-walled and single-walled microspheres are shown in Figure 3. The release was examined in phosphate buffer saline (PBS) solution (pH = 7.4). A significant reduction in the initial burst release of lysozyme was achieved for double-walled microspheres prepared in different formulations when compared to single-walled formulations. It can be observed that double-walled microspheres of formulations LF1 and LF3 released 15.43 ± 1.86% and 14.43 ± 2.56% of encapsulated lysozyme, respectively, within the first 6 h (Figure 3a). Increasing the mass ratio of Glu-PLGA to PLGA from 1:1 to 2:1 in formulations LF2 and LF4 showed further reduction of lysozyme released to 11.14 ± 2.78% and 10.14 ± 1.97%, respectively (Figure 3a). A decrease in the initial burst release of hydrophilic drugs with an increasing mass ratio of the component polymers was observed for double-walled microspheres prepared by other researchers [23,24]. In a study of Zheng (2009), the initial burst release of 5-FU from double-walled microspheres made of PLGA 80/20 and PLGA 75/25 polymers in a mass ratio of 1:1 was 16.7 ± 2.1%, whereas increasing the mass ratio of the component polymers up to 2:1 made a further reduction in the initial burst release to 4.2 ± 0.9% [23]. The drug-free outer layer of double-walled microspheres acts as a barrier, which inhibits the rapid diffusion of hydrophilic lysozyme localized in the core of the microspheres. With the increasing mass ratio, as the microspheres size increases, the distance between the core of the microspheres and the release medium also increased, which resulted in a low initial burst. Moreover, as our prepared double-walled microspheres were non-porous and had smooth surfaces as compared to single-walled microspheres, the water penetration rate inside the microspheres through the pre-existing pores or channels was probably reduced, which resulted in a low initial burst.

Single-walled microspheres prepared in different formulations showed a high initial burst release. It can be noticed in Figure 3b that about 55% of the loaded lysozyme was released from the single-walled formulations of LF5, LF7, and LF8 within the first 6 h of incubation. Similar results of very high initial burst release of lysozyme were observed in previous studies [31,32]. In a study of Diwan and Park (2001), about 50% of lysozyme was released from PLGA microspheres after 1 h of incubation [31]. In the present study, with the increasing polymer concentration, a slightly reduced initial burst of 35.32 ± 3.15% (released within 6 h) was observed in another single-walled formulation (LF6). The very high initial burst release of single-walled formulations observed is due to the rapid diffusion of lysozyme through the pores to the incubation medium. Single-walled microspheres prepared in the formulations LF5, LF7, and LF8 were highly porous and lysozyme molecules placed at these pores could easily diffuse out due to water penetration after incubation, which resulted in a higher initial burst [33]. The reduced surface porosity of formulation LF6 controlled the high initial burst release only scarcely.

Figure 3c shows 70 days in vitro release profiles of lysozyme from double-walled and single-walled microspheres formulations. Double-walled microspheres exhibited 21.98 ± 3.42% and 18.65 ± 2.52% of lysozyme released after day 1 from formulations LF1 and LF3, respectively. This was followed by a very slow release and subsequent incomplete release until day 70. At this point, only 42.97 ± 2.48% and 41.74 ± 2.03% of lysozyme was released from formulations LF1 and LF3, respectively. With increasing the mass ratio of Glu-PLGA to PLGA in formulations LF2 and LF4 showed similar results of a very slow release of lysozyme after day 1, and subsequently an incomplete release until day 70. During this period, only 32.13 ± 3.41% and 30.97 ± 2.38% of lysozyme was released from formulations LF2 and LF4, respectively. Single-walled microspheres prepared in formulation LF6 showed 43.20 ± 3.29% of lysozyme released after day 1 which was followed by slow release, reaching 69.21 ± 2.69% of the encapsulated lysozyme at day 70. Importantly, the release profiles of single-walled and double-walled microspheres formulations showed approximately 4–8% of lysozyme released between day 35 and 70. Therefore, it can be summarized that there was no significant release of lysozyme after day 35 from all of the formulations as indicated by a plateau. Similar results of the initial burst release, followed by very slow and incomplete releases of encapsulated lysozyme from PLGA microspheres have been observed in previous studies [12,31]. It has been reported by Jiang et al. (2002) that only 27% of the encapsulated lysozyme was released from PLGA microspheres within 70 days [12]. In another study, about 40% of the encapsulated lysozyme was released after 1 h of incubation and the release reached to about 70% of the loaded amount at day 83 [31]. Several reasons for the incomplete release of encapsulated proteins from PLGA microspheres have been described in the literature. Particularly, the covalent or non-covalent aggregation of the entrapped protein during microspheres fabrication and in vitro release studies, protein adsorption on the surface of PLGA matrix, interactions between encapsulated proteins and PLGA, and chemical degradation of encapsulated proteins are the most common issues that affect protein release kinetics [31,34,35,36].

To investigate the incomplete release above, the remaining insoluble residues present in the micro centrifuge tubes after 70 days in vitro release studies were centrifuged, washed three times with distilled water, and freeze-dried over night. The freeze-dried residues were then dissolved in DMSO and 0.5% (*w*/*v*) SDS containing 0.05 M NaOH solutions. The protein content in the DMSO digested solution was then quantified by a micro BCA protein assay kit. At the same time, the enzymatic activity of the released lysozyme was determined.

Table 3 shows that about 12–21% of the encapsulated lysozyme was extracted from the 70 days remaining residue of different formulations. A large portion of the encapsulated lysozyme (40–60%) was not recovered from the double-walled formulations. The incomplete recovery might be due to further aggregation or denaturation of protein during the in vitro release experiments where protein was exposed to an acidic environment due to polymer erosion [32,34]. It has been reported that the aggregated or denatured protein molecules remain in the microspheres as insoluble residue, which cannot be quantified by the BCA method [29]. As can be seen in Table 3, lysozyme released from the double-walled and single-walled microspheres was fully bioactive. Therefore, it can be said that microencapsulation and in vitro release process preserved the bioactivity of the released lysozyme.

### 3.6. Stabilization of Lysozyme during Emulsification

The in vitro release studies of double-walled and single-walled microspheres showed an incomplete release of the encapsulated lysozyme after a particular time period of microspheres degradation, either due to lysozyme aggregation or adsorption on to the strong hydrophobic surfaces of the PLGA matrix. It has already been mentioned that the primary emulsification step of making w_1_/o emulsion is the most detrimental for protein stability. Maximum protein deactivation takes place at the aqueous/organic interface, resulting in protein aggregation and adsorption. In order to protect lysozyme from the aqueous/organic interface-induced adsorption, aggregation, and denaturation, several protein stabilizing excipients such as sucrose, trehalose, glycerol, cyclodextrins, Tween 20, Tween 80, bovine serum albumin, rat serum albumin, and partially hydrolysed PVA have been co-dissolved with proteins prior to the primary emulsification step of microspheres fabrication in the previous studies [29,35,36,37,38]. It has been reported that most of the excipients lower the aqueous/organic interfacial tension during primary emulsification and occupy the available sites of the interface. As a result, protein molecules are shielded from the contact of water/organic solvent interface, which inhibits protein adsorption [36]. Moreover, the incorporation of sugars (sucrose, trehalose) in the emulsification step not only protects proteins from surface induced aggregation, but also stabilizes proteins from dehydration-induced structural changes during lyophilization. In a study, co-encapsulation of serum albumin during emulsification improved the release kinetics of lysozyme from PLGA microspheres [37]. But, the quantification of lysozyme in the in vitro released sample might be more complicated in presence of an additional protein as reported [37]. In another study, it has been found that the addition of non-ionic surfactants (Tween 20 and Tween 80) had no effect on improving the release kinetics of lysozyme from PLGA microspheres; whereas, the incorporation of PVA had a significant effect on improving lysozyme recovery from PLGA microspheres [36].

In these circumstances, attempts were made to improve the in vitro release profiles of lysozyme from double-walled microspheres by the incorporation of PVA in the internal aqueous phase and trehalose in the external aqueous phase. Lysozyme dissolved in 1% aqueous PVA (*w*/*v*) solution was used as an internal aqueous phase (w_1_), whereas 2.5% aqueous trehalose (*w*/*v*) solution was used for the preparation of an external aqueous phase (w_2_). The formulation and process variables were the same as double-walled microspheres were prepared in formulation LF1.

#### 3.6.1. Excipients Effect on Microspheres Size

Table 4 shows that the mean particle size of double-walled microspheres varied slightly from 1.64 ± 0.58 to 4.34 ± 1.20 µm with the addition of excipients in the internal and external aqueous phase. The addition of 1% PVA (*w*/*v*) in the internal aqueous phase did not show a marked difference in the mean particle size as compared to microspheres prepared free from PVA in the internal aqueous phase. In contrast, a statistically significant increase (*p* < 0.05) in the mean particle size was observed when microspheres were prepared with the incorporation of 2.5% trehalose (*w*/*v*) in the internal aqueous phase. With the addition of trehalose in the internal aqueous phase, the w_1_/o emulsion droplets might be destabilized due to the inward diffusion of water molecules from the external to the internal aqueous phase because of high osmotic pressure. This phenomenon leads to swelling and disrupting of the internal emulsion droplets, which favors coalescence of the micro droplets, and results in the formation of microspheres with a slightly larger size. It has been reported that the presence of highly water soluble excipients (e.g., sugar, salt) in the internal aqueous phase at higher concentrations forms highly porous and even ruptured microspheres [39]. It was observed that the addition of 1% PVA (*w*/*v*) in the internal aqueous phase and 2.5% trehalose (*w*/*v*) in the external aqueous phase interestingly reduced the microspheres size to 2.61 ± 0.50 µm as compared to the addition of trehalose in the internal aqueous phase alone. This might be due to the increase in viscosity of the external aqueous phase as trehalose was incorporated in the external continuous phase. The increased viscosity of the external aqueous phase might inhibit the diffusion of the external aqueous phase to internal aqueous phase and resulting in a balancing of osmotic pressure between the two phases. This balancing of osmotic pressure could stabilize the emulsion droplets against coalescence and result in reduced microspheres size. In a study, the addition of glucose in the external aqueous phase showed a remarkable decrease in the microspheres size from 200 µm to 45 um [40]. The average particle size of microspheres prepared without glucose in the external aqueous phase was 200 µm, whereas the addition of 2.5% glucose (*w*/*v*) in the external aqueous phase reduced the particle size to 85 µm and a gradual decrease in the microspheres size was observed with increasing the concentration of glucose solution. In the above studies, the increased viscosity of the external aqueous phase due to the addition of glucose was considered to be a reason of producing microspheres with a reduced particle size.

#### 3.6.2. Excipients Effect on Surface Morphology

**Figure 4:**
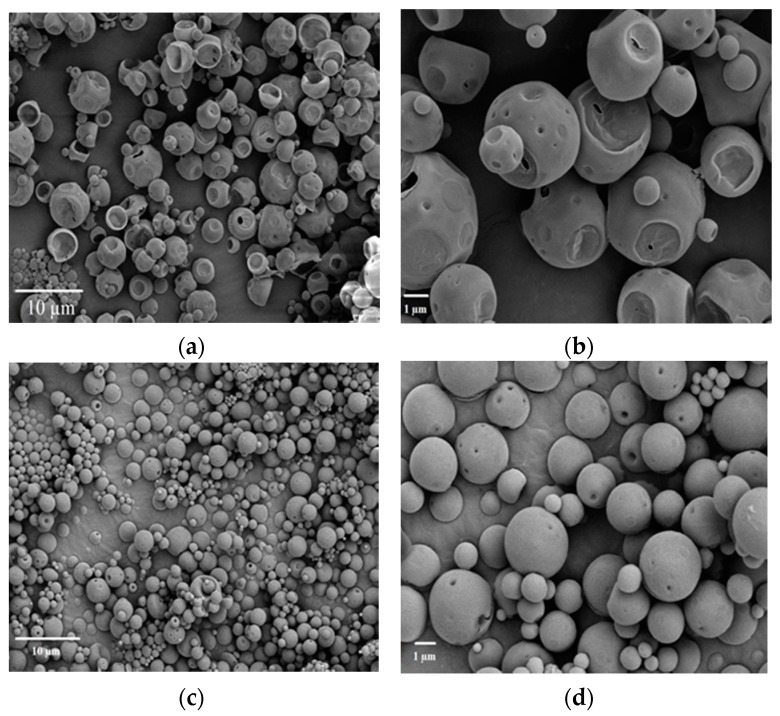
Field-emission scanning electron microscope (FE-SEM) images of lysozyme loaded double-walled microspheres prepared with 2.5% trehalose (*w*/*v*) in internal aqueous phase at different resolution: (**a**) 2000× mag; (**b**) 6000× mag and double-walled microspheres prepared with 1% PVA (*w*/*v*) in internal aqueous phase; and, 2.5% trehalose (*w*/*v*) in external aqueous phase at different resolution: (**c**) 2000× mag; (**d**) 6000× mag.

Figure 4 demonstrated the excipients effect on the morphologies of lysozyme loaded double-walled microspheres. FE-SEM images revealed in Figure 4a,b that porous and squeezed shape double-walled microspheres were formed with the addition of 2.5% trehalose (*w*/*v*) in the internal aqueous phase. In contrast, the external morphologies of double-walled microspheres prepared with 1% PVA (*w*/*v*) in internal aqueous phase and 2.5% (*w*/*v*) trehalose in the external aqueous were spherical in shape and had smooth and nonporous surfaces (Figure 4c,d). As discussed earlier, the incorporation of 2.5% (*w*/*v*) trehalose in the internal aqueous phase leads to an inward diffusion of the external aqueous phase to internal aqueous phase for balancing of the osmotic pressure between the two phases, which resulted in swelling and disrupting of the internal droplets and a loss of drug molecules being encapsulated. The water diffusion channels might appear as pores after solidification of the microspheres. Therefore, the porous and squeezed surfaces of the microspheres prepared with 2.5% trehalose (*w*/*v*) in the internal aqueous phase might be a result of the inward diffusion of water molecules from the external to the internal aqueous phase. The water diffusion might be inhibited due to the incorporation of 1% PVA (*w*/*v*) in the internal aqueous phase and 2.5% trehalose (*w*/*v*) in the external aqueous because of the balancing of osmotic pressure, which resulted in spherical, dense, and nonporous microspheres. 

#### 3.6.3. Excipients Effect on Lysozyme Encapsulation Efficiency

The incorporation of 2.5% trehalose (*w*/*v*) solution alone in the internal aqueous phase showed a statistically significant decrease (*p* < 0.05) in encapsulation efficiency when compared to other excipients containing formulations (Table 4). During emulsification and microspheres hardening, the inward diffusion of water molecules from external to the internal aqueous phase could destabilize the w_1_/o/o/w_2_ emulsion droplets, which could trigger the partitioning of lysozyme molecules from internal to the external aqueous phase, and result in a low encapsulation efficiency. With the addition of 1% PVA (*w*/*v*) in the internal aqueous phase and 2.5% trehalose (*w*/*v*) in the external aqueous phase, no water molecules might diffuse from the external to the internal phase, which contributes to the formation of spherical, dense, and absolutely nonporous and compact shape microspheres, and result in a high encapsulation efficiency.

#### 3.6.4. Excipients Effect on In Vitro Lysozyme Release Profiles

**Figure 5:**
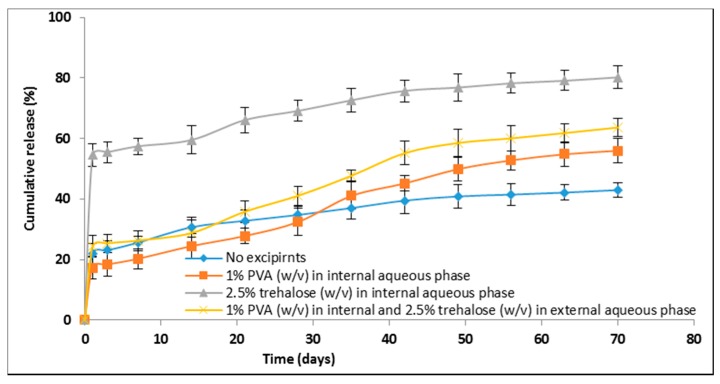
In vitro release profiles of lysozyme from double-walled microspheres prepared together with excipients and without excipients in the internal and external aqueous phase during preparation. The data are presented as mean ± SD (*n* = 3).

The in vitro release profiles of lysozyme from double-walled microspheres prepared with excipients (trehalose, PVA) in the internal and external aqueous phase are shown in Figure 5. It has been observed that the release profiles of microspheres prepared with excipients were significantly different from the release profile of microspheres prepared without excipients. The microspheres prepared without trehalose or PVA either in the internal or external aqueous phase showed 21.99 ± 3.41% of lysozyme released after day 1, and subsequently slow and incomplete release until day 70. During this time period, the cumulative release of lysozyme was only 42.97 ± 2.48%. In contrast, microspheres prepared with the addition of trehalose in the internal aqueous phase showed as high as 54.54 ± 3.84% of lysozyme released for the first day and the release was reached to 81.21 ± 4.22% after day 70. This statistically significant increase in lysozyme released might be due to the formation of pores or channels during the microspheres preparation caused inward diffusion of water molecules from external to the internal aqueous phase of the emulsion droplets.

The incorporation of 1% PVA (*w*/*v*) in the internal aqueous phase slightly reduced the day 1 release to 17.32 ± 3.84%, whereas the cumulative release was increased to 55.97 ± 4.15% after day 70. Among the excipients-containing formulations, a more sustained release of lysozyme was achieved with the incorporation of 1% PVA (*w*/*v*) in the internal aqueous phase and 2.5% trehalose (*w*/*v*) in the outer aqueous phase during microspheres preparation. In this case, a tri-phasic release profile was observed. Initially, 24.32 ± 3.57% of lysozyme was released within the first 24 h, then the release was continued very slowly until day 14, and subsequently a faster and sustained release was observed until day 70. Between day 1 and 70, the highest amount of 39% lysozyme was released from the microspheres prepared with 1% PVA (*w*/*v*) in the internal aqueous phase and 2.5% trehalose (*w*/*v*) in the external aqueous phase. This might be due to the presence of PVA in the internal aqueous phase that could stabilize lysozyme against aqueous/organic interface induced denaturation and aggregation. The incorporation of PVA in the internal aqueous phase improves lysozyme stability probably by competing with lysozyme adsorption at large water/organic interface [35,36]. Moreover, the addition of trehalose in the external aqueous phase might stabilize lysozyme against dehydration-induced denaturation and aggregation during freeze-drying [41]. Therefore, the presence of these two excipients in the fabrication process might increase the total percentage of stabilized lysozyme in the microspheres, which is assumed to be a reason of improved lysozyme release. Although the in vitro release profiles showed an improved lysozyme release (~64%) within 70 days, the complete release was not achieved during this time frame. This might be due to the inherent stability problem of proteins including shear stress denaturation during homogenization [31], moisture induced denaturation during incubation [37,38,42], and pH induced denaturation during polymer erosion [43]. Therefore, the above issues need to be solved for the complete release of lysozyme.

### 3.7. Lysozyme Stability during Encapsulation and In Vitro Release

**Figure 6:**
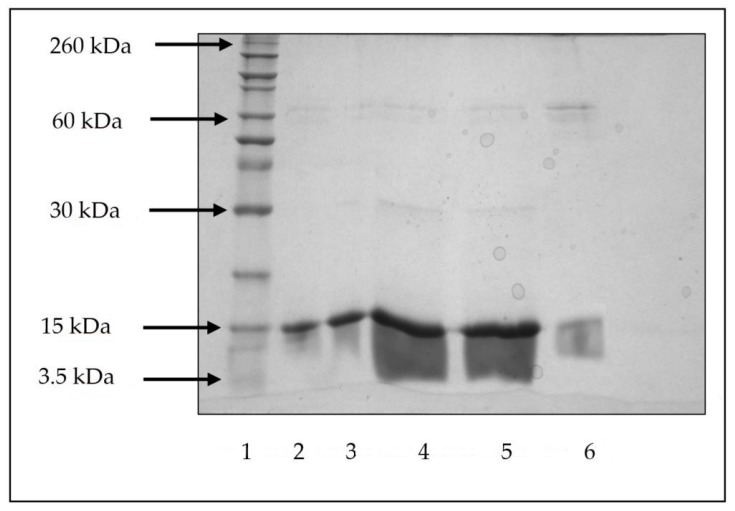
Polyacrylamide Gel Electrophoresis (SDS-PAGE) of lysozyme extracted and released from double-walled microspheres. Lane 1: standard molecular weight marker (*M*_w_ range: 3.5–260 kDa); Lane 2: fresh lysozyme standard; Lane 3: lysozyme extracted from microspheres prepared without excipients; Lane 4: day 1 released lysozyme from microspheres prepared without excipients; Lanes 5–6: day 1 and day 42 released lysozyme from microspheres prepared with 1% PVA (*w*/*v*) in internal aqueous phase and 2.5% trehalose (*w*/*v*) in external aqueous phase, respectively.

SDS-PAGE of extracted and released lysozyme was performed under non-reducing condition and the results are shown in Figure 6. Fresh, extracted, and released supernatants exhibited strong bands of lysozyme monomer (around 15 kDa) along with weak dimeric (around 30 kDa) and multimeric (around 60 kDa) bands of aggregated species. Due to a low lysozyme concentration, the released sample on day 42 exhibited a low intense band when compared to released sample on day 1. The presence of weak dimeric and a multimeric band of all samples proves that a small fraction of fresh, extracted, and released lysozyme underwent aggregation. Similar results of protein aggregation have been observed in previous studies [26,44]. Since, the SDS-PAGE was performed under non-reducing conditions, the aggregated bands might be a result of intramolecular disulfide-coupled protein aggregates [45]. Moreover, it is clear from the gel that fresh, as well as extracted and released, lysozyme molecules underwent fragmentation to some extent in addition to aggregation. The presence of hydrolyzed fragments of fresh lysozyme in the gel implies that the stability problem of lysozyme in solution is an inherent problem.

## 4. Conclusions

In this study, a model protein-lysozyme encapsulated in double-walled microspheres comprising of Glu-PLGA and PLGA has been fabricated successfully using a modified w_1_/o/o/w_2_ emulsion solvent evaporation technique. The prepared microspheres were dense, spherical in shape, and had smooth surfaces when Glu-PLGA and PLGA mass ratio was 1:1. In contrast, single-walled microspheres were irregular in shape and highly porous. A significant reduction in initial burst was achieved by the production of double-walled microspheres as compared to single-walled microspheres. Double-walled microspheres exhibited approximately 20% of lysozyme released after day 1, which was followed by a very slow release and subsequent incomplete release until day 70. During this time frame about 40% of lysozyme was released. Moisture induced aggregation and denaturation of protein during microspheres fabrication, and in vitro release studies are considered as a reason of incomplete lysozyme release. An improved lysozyme release was observed when double-walled microspheres were fabricated together with excipients (PVA and trehalose). The incorporation of 1% PVA (*w*/*v*) in the internal aqueous phase and 2.5% (*w*/*v*) trehalose in the external aqueous phase showed approximately 24% of lysozyme release from double-walled microspheres after day 1. The release continued in a sustained manner that reached up to approximately 64% after day 70. Therefore, double-walled microspheres comprising of Glu-PLGA and PLGA provided an improved lysozyme release profiles when excipients (PVA and trehalose) were co-encapsulated during microspheres fabrication.

## Figures and Tables

**Table 1:** Polymer and solvent used for the preparation of lysozyme loaded microspheres in different formulations.

Formulation Code	Method	Glu-PLGA * (Low *M*_W_) (mg)	Glu-PLGA ** (High *M*_W_) (mg)	PLGA (mg)	DCM (mL)	EA (mL)
LF1	w_1_/o/o/w_2_	50	-	50	1	1
LF2	w_1_/o/o/w_2_	100	-	50	1	1
LF3	w_1_/o/o/w_2_	-	50	50	1	1
LF4	w_1_/o/o/w_2_	-	100	50	1	1
LF5	w_1_/o/w_2_	50	-	-	1	-
LF6	w_1_/o/w_2_	100	-	-	1	-
LF7	w_1_/o/w_2_	-	50	-	1	-
LF8	w_1_/o/w_2_	-	-	50	1	-

* Number average molecular weight, *M*_n_ = 15,000;

** Number average molecular weight, *M*_n_ = 50,000.

**Table 2:** Characteristics of lysozyme loaded microspheres prepared in different formulations.

Formulation Code	Mean Particle Size (µm ± SD)	Span ± SD	Actual Drug Loading (% ± SD)	Encapsulation Efficiency (% ± SD)	Microspheres Yield (%)
LF1	1.64 ± 0.58	1.01 ± 0.08	2.13 ± 0.10	71.04 ± 3.08	58.85 ± 4.15
LF2	7.79 ± 1.03	1.34 ± 0.12	2.49 ± 0.12	82.84 ± 3.89	63.54 ± 3.28
LF3	2.65 ± 0.45	1.14 ± 0.05	2.17 ± 0.11	72.15 ± 3.54	61.28 ± 3.72
LF4	8.04 ± 1.21	1.31 ± 0.07	2.52 ± 0.14	83.92 ± 4.66	67.36 ± 3.86
LF5	6.32 ± 1.07	1.27 ± 0.13	1.59 ± 0.11	51.67 ± 3.56	64.18 ± 4.32
LF6	7.18 ± 1.38	1.38 ± 0.10	1.85 ± 0.13	62.03 ± 4.15	66.45 ± 3.55
LF7	5.73 ± 1.21	1.22 ± 0.12	1.65 ± 0.09	53.88 ± 2.97	62.40 ± 4.14
LF8	5.20 ± 1.10	1.16 ± 0.11	1.62 ± 0.12	52.23 ± 3.90	63.12 ± 3.37

SD = Standard deviation, *n* = 3.

**Table 3:** Lysozyme specific bioactivity and recovery from double-walled and single-walled microspheres prepared in different formulations.

Formulation Code	Lysozyme Released from the Microspheres after 70 Days (% ± SD )	Lysozyme Extracted from the Insoluble Residues (% ± SD)	Recovery (% ± SD)	Specific Bioactivity of the Released Lysozyme (% ± SD)
LF1	42.97 ± 2.48	18.54 ± 3.65	61.51 ± 3.73	100.78 ± 4.90
LF2	32.13 ± 3.41	13.98 ± 4.38	46.11 ± 1.16	101.43 ± 4.60
LF3	41.74 ± 2.02	19.75 ± 3.21	61.48 ± 4.37	99.54 ± 3.25
LF4	30.97 ± 2.38	12.14 ± 3.53	43.10 ± 1.21	99.14 ± 5.56
LF6	69.21 ± 2.69	21.02 ± 3.11	90.23 ± 4.92	101.15 ± 4.08

SD = Standard deviation, *n* = 3.

**Table 4:** Effect of excipients in internal aqueous phase and external aqueous phase on particle size, span value and encapsulation efficiency for lysozyme loaded double-walled microspheres.

Excipients Present in Internal Aqueous Phase (*w*/*v*)%	Excipients Present in External Aqueous Phase (*w*/*v*)%	Mean Particle Size (µm ± SD)	Span ± SD	Encapsulation Efficiency (± SD)
-	-	1.64 ± 0.58	1.01 ± 0.08	71.04 ± 3.08
1% PVA	-	1.73 ± 0.53	1.12 ± 0.05	75.65 ± 4.62
2.5% trehalose	-	4.34 ± 1.20	1.20 ± 0.12	54.15 ± 3.80
1% PVA	2.5% trehalose	2.61 ± 0.50	1.09 ± 0.07	82.52 ± 3.28

SD = Standard deviation, *n* = 3.
